# Hydroxyapatite Double Substituted with Zinc and Silicate Ions: Possibility of Mechanochemical Synthesis and In Vitro Properties

**DOI:** 10.3390/ma16041385

**Published:** 2023-02-07

**Authors:** Svetlana V. Makarova, Natalia V. Bulina, Yuliya A. Golubeva, Lyubov S. Klyushova, Natalya B. Dumchenko, Svetlana S. Shatskaya, Arcady V. Ishchenko, Mikhail V. Khvostov, Dina V. Dudina

**Affiliations:** 1 Institute of Solid State Chemistry and Mechanochemistry, Siberian Branch of the Russian Academy of Sciences, 630090 Novosibirsk, Russia; 2 Nikolaev Institute of Inorganic Chemistry, Siberian Branch of the Russian Academy of Sciences, 630090 Novosibirsk, Russia; 3 Department of Natural Sciences, Novosibirsk State University, 630090 Novosibirsk, Russia; 4 Institute of Molecular Biology and Biophysics of Federal State Budget Scientific Institution “Federal Research Center of Fundamental and Translational Medicine” (IMBB FRC FTM), 630060 Novosibirsk, Russia; 5 State Research Center of Virology and Biotechnology VECTOR, Federal Service for Surveillance in Consumer Rights Protection and Human Well-being, 630559 Koltsovo, Russia; 6 G. K. Boreskov Institute of Catalysis, Siberian Branch of Russian Academy of Sciences, 630090 Novosibirsk, Russia; 7 Vorozhtsov Novosibirsk Institute of Organic Chemistry, Siberian Branch of the Russian Academy of Sciences, 630090 Novosibirsk, Russia; 8 Lavrentyev Institute of Hydrodynamics, Siberian Branch of the Russian Academy of Sciences, 630090 Novosibirsk, Russia

**Keywords:** hydroxyapatite, co-substitution, doping, zinc, silicone, cytotoxicity, biocompatibility

## Abstract

In this study, the mechanochemical synthesis of substituted hydroxyapatite (HA) containing zinc and silicon ions having a chemical formula of Ca_10−x_Zn_x_(PO_4_)_6−x_(SiO_4_)_x_(OH)_2−x_, where x = 0.2, 0.6, 1.0, 1.5, and 2.0, was carried out. The synthesized materials were characterized by powder X-ray diffraction, Fourier transform infrared spectroscopy, transmission electron microscopy, and inductively coupled plasma spectroscopy. We found that HA co-substituted with zinc and silicate formed up to x = 1.0. At higher concentrations of the substituents, the formation of large amounts of an amorphous phase was observed. The cytotoxicity and biocompatibility of the co-substituted HA was studied in vitro on Hek293 and MG-63 cell lines. The HA co-substituted with zinc and silicate demonstrated high biocompatibility; the lowest cytotoxicity was observed at x = 0.2. For this composition, good proliferation of MG-63 osteoblast-like cells and an increased solubility compared with that of HA were detected. These properties allow us to recommend the synthesized material for medical applications, namely, for the restoration of bone tissue and manufacture of biodegradable implants.

## 1. Introduction

The chemical composition of hydroxyapatite (HA), Ca_10_(PO_4_)_6_(OH)_2_, is close to that of human bone. Synthetic HA is actively used in medicine [1]. In dentistry, it is used to fill bone voids after a tooth has been extracted and in the treatment of pulpitis [2]. In maxillofacial surgery, HA can form bone implants or bioactive coatings on metal implants to improve and accelerate the osseointegration process [3]. However, because of a low mechanical strength of HA, it cannot be used alone to form implants of the musculoskeletal system, as the latter must withstand high stresses. HA is a component of many toothpastes and powders, providing protection from the formation of plaque and calculus [4,5]. HA’s microporous structures act as drug delivery agents and are used for the controlled release of antineoplastic drugs and antibiotics [6,7].

One of the main features of HA is its ability to form homo- and heterovalent ion-substituted compounds [8]. The hydroxyl groups in the HA structure can be replaced by Cl^−^, F^−^, and CO_3_^2−^ anions [9,10]. The phosphate ions can be replaced by CO_3_^2−^, SeO_3_^2−^, and SiO_4_^4−^; calcium cations can be replaced by Na^+^, Zn^2+^, La^3+^, and Sr^2+^ cations [11,12]. The structure of HA allows for multi-ion substitution: several cations and/or anions of different nature can be substituted into its lattice [13]. The introduction of even a small amount of certain ions can improve the biological, mechanical, and physicochemical properties of HA. For example, the introduction of silicate ions has a positive effect, enhancing the osseointegration of the material and increasing its biocompatibility [14]. Two of the key problems in surgery are implant rejection and infection. For protection, an antibiotic has to be introduced into the body or directly at the site of implantation. The concentration of the antibiotic must be high enough to kill the infection. At the same time, the antibiotic must not cause harm to the body. Therefore, increasing the antibacterial properties of the implanted material is an important task. For example, the introduction of zinc ions into the HA structure makes it possible to obtain materials with antibacterial properties [15,16].

HA can be obtained by both liquid-phase and solid-state methods [17]. The mechanochemical method is a promising solid-state method for obtaining unsubstituted and substituted HA. The main advantages of this method include the rapid formation of a single-phase HA product, the possibility of introducing substituent ions, and the absence of solvents in the synthesis. The latter eliminates the need to maintain the pH of the reaction mixture [18]. Friederichs et al. [19] reported the formation of HA containing zinc and silicate ions by precipitation from aqueous solution followed by thermal treatment at 1100 °C. However, the properties of this material have not been studied.

The aim of this study was to obtain HA co-substituted with zinc and silicate (ZnSi-HA) via soft mechanochemical synthesis without the use of additional heat treatment and to study the biological properties of the resultant materials.

## 2. Materials and Methods

### 2.1. Synthesis of Substituted HA

A series of ZnSi-HA samples with a general chemical formula of Ca_10−x_Zn_x_(PO_4_)_6−x_(SiO_4_)_x_(OH)_2−x_ was obtained by mechanochemical synthesis (MS) in a high-energy planetary ball mill (AGO-2 model). The synthesis was carried out in water-cooled steel vials filled with 8 mm steel balls. The rotation speed of the vials was 1200 rpm. The milling time was 5, 10, 15, 20, 25, 30, or 40 min. To prevent contamination of the product of synthesis by the milling debris, the walls of vials and the surface of the balls were lined with the reaction mixture (for that, the reaction mixture was milled in the vials for 0.5 min). The initial reagents were freshly calcined calcium oxide CaO, calcium hydrophosphate CaHPO_4_, silicon oxide SiO_2_·0.7 H_2_O, and zinc dihydrogen phosphate dihydrate Zn(H_2_PO_4_)_2_·2H_2_O. The ratio of the reagents was taken in accordance with equations given in Table 1, assuming the substitution of zinc ions for calcium ions and silicate ions for the phosphate groups. All synthesized samplers were fine, white powders.

The analytical and in vitro investigations, except for the MG63 cell proliferation analysis, were performed on powdered samples. For the latter, pellets with a diameter of 5 mm and a mass of 0.06 g were prepared. The pellets were obtained by pressing the powder material in a steel die at a pressure of 500 MPa.

### 2.2. Analysis of Synthesized Compounds

The obtained powders were analyzed with several analytical methods.

X-ray diffractions (XRD) patterns were recorded on a D8 Advance powder diffractometer (Bruker, Karlsruhe, Germany) with Bragg–Brentano geometry using Cu-Kα radiation. The X-ray phase analysis of the compounds was carried out using the ICDD PDF-4 database (2011). The unit cell parameters, crystallite size, and amorphous phase content were determined by the Rietveld method in Topas 4.2 software (Bruker, Karlsruhe, Germany).

The Fourier transform infrared (FTIR) spectra of the powders were recorded on an Infralum FT-801 spectrometer (Simex, Novosibirsk, Russia). The specimens were prepared by the KBr pellet method.

Transmission electron microscopy (TEM) images were obtained using a JEM-2200FS microscope (JEOL Ltd., Akishima, Japan). High-resolution TEM (HRTEM) was also carried out. Energy-dispersive X-ray microanalysis (EDX) of the samples was performed by a four-segment Super-X detector in scanning dark-field mode with the maps of distributions of elements constructed with the characteristic lines of the spectrum from each point in the analyzed region. The samples for the TEM examination were dispersed by ultrasonication and deposited from an alcohol-based suspension on an aluminum substrate.

The elemental analysis of the synthesized materials was carried out by inductively coupled plasma atomic emission spectroscopy (ICP-AES) using an iCAP 6500 (Thermo Scientific, Waltham, MA, USA) spectrometer. The powder samples were dissolved in aqua regia (50 mg per 2 mL) upon heating. The resultant solutions were analyzed. The plasma was observed axially to obtain the best possible sensitivity. The background corrected signals were used for the quantitative analysis. 

### 2.3. In Vitro Investigations

The cytotoxicity and biocompatibility of the obtained materials were studied on human embryonic kidney Hek293 cell line (ATCC, Manassas, VA, USA) and human-osteosarcoma-derived MG-63 cell line (Center for Vertebrate Cell Culture Collection, St. Petersburg, Russia), respectively.

The viability of the Hek293 cell line was evaluated with Hoechst 33342/propidium iodide staining by the standard method described in [20]. The cells were seeded on 96-well plates at 5 × 10^3^ cells per well and cultured in Iscove’s modified Dulbecco’s medium (IMDM, pH = 7.4) supplemented with 10% fetal bovine serum in a CO_2_ incubator at 37 °C. After 24 h, the cells were treated with water suspension of the synthesized powders at concentrations of 0.01–50 mg/mL for 48 h. For identifying the live, apoptotic, and dead cells, the treated and control cells were stained with a mixture of fluorescent dyes: Hoechst 33,342 (Sigma-Aldrich, St. Louis, MA, USA) and propidium iodide (Invitrogen, Waltham, MA, USA), for 30 min at 37 °C. IN A Cell Analyzer 2200 (GE Healthcare, Chicago, IL, USA) was used to perform four fields per well of automatic imaging in bright-field and fluorescence channels. By means of IN Cell Investigator image analysis software (version 1.5, GE Healthcare, Chicago, IL, USA) in accordance with the morphological changes, the cells were classified as live and apoptotic cells. All data shown are the mean of three wells. The quantitative data are expressed as the mean ± standard deviation (SD).

The MG-63 cells were cultured in 96-well plates (Costar, Washington, DC, USA). DMEM (State Research Center of Virology and Biotechnology VECTOR, Koltsovo, Russia) supplemented with 1–5% fetal bovine serum (Gibco, Grand Island, NY, USA) served as the growth medium. For the MTT assay, MG63 cells were attached to the plate bottom; a sterile synthesized powder was added to the medium to a concentration of 2.5 × 10^−5^ g/L in each well. This concentration was selected as the optimal for optical-density measurement in a 3-(4,5-dimethylthiazol-2-yl)-2,5-diphenyl-2H-tetrazolium bromide (MTT) assay. After 96 h, the cell viability was assessed with the MTT assay. For this purpose, 5 μL of an MTT solution (Sigma, St. Louis, MA, USA) was added into the wells and incubated for 4 h. At the end of the incubation, 100 μL of dimethyl sulfoxide (Vecton, Saint Petersburg, Russia) was added, and the cell viability was determined by means of the color intensity of the resultant formazan solution. The optical density was measured on a Tecan Sunrise microplate reader (Tecan, Grödig, Austria) at a wavelength of 492 nm.

For the bone cell proliferation assessment, MG-63 cells were seeded on the pellet samples and kept in the growth medium for 7 days. The cell concentration in the growth medium was 2 × 10^3^ cells per well. The cells on the pellet samples were fixed with 2% glutaraldehyde buffer (pH 7.4) at room temperature for 12 h, subsequently washed three times with HEPES (0.1 M) buffer, and dehydrated by increasing the concentration of alcohol (50%, 70%, 95%, and 100%) twice for 10 min each. Finally, the samples were chemically dried (4 steps with different ratios of alcohol to HMDS) and sputter-coated with gold under a vacuum. The cell morphology was examined using scanning electron microscopy on a TM-1000 Tabletop microscope (HITACHI, Tokyo, Japan).

To evaluate the bioresorption, the solubility of the synthesized powders in water was studied. The powders were placed in distilled water at a concentration of 0.2 g/mL and kept for 1, 3, and 5 days. After the specified time, the suspensions were filtered. The quantitative determination of ions in the mother liquor was carried out on an AA-280FS atomic absorption spectrometer (Varian, Inc., Palo Alto, Santa Clara, CA, USA). The work was carried out under a hollow cathode lamp current of 5 mA, a flame of air-acetylene oxidative, a wavelength of 213.9 nm, a monochromator slit width of 1.0 nm, and optimal working area of 0.01–2 ppm.

## 3. Results and Discussion

### 3.1. Determination of the Optimal Conditions of the Mechanochemical Synthesis

One of the main parameters of mechanochemical synthesis is the time of treatment. If the process is not long enough, there will be incomplete conversion of the components of the initial mixture into the target product, and the reactants will be present in the mixture. When the time of treatment is excessively long, complete or partial decomposition of the reaction product is possible. Therefore, determining the optimal time for obtaining the target product is a primary task in mechanochemical synthesis. To determine the optimal synthesis time, the reaction mixtures (Table 1) were treated in a planetary ball mill for 5–40 min.

**Figure 1:**
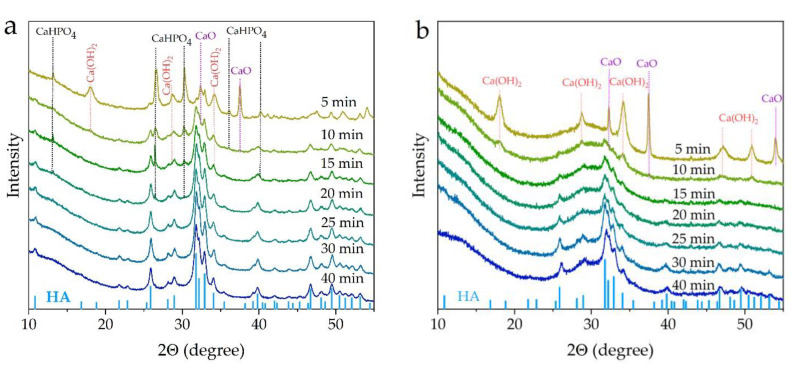
XRD patterns of 1.0-ZnSi-HA (**a**) and 2.0-ZnSi-HA (**b**) samples after different mechanical treatment times.

Figure 1 shows the XRD patterns of the reaction mixtures for samples with high contents of the substituent ions, namely 1.0-ZnSi-HA and 2.0-ZnSi-HA, treated in the mill for different times. The most intense reflection of the HA phase appeared after 5 min of mechanical treatment of 1.0-ZnSi-HA. However, the reflections of the initial reagents were retained in the mixture for up to 20 min of treatment. In the 2.0-ZnSi-HA sample, due to the high concentration of hydrate water (Table 1), the reaction proceeded faster—the reflections of the initial reagents were absent already after 15 min of treatment. It should be noted that this sample contained a large amount of an amorphous phase (22–40° 2θ), which remained even after 40 min of treatment. This may have been due to a larger amount of water being released during the first seconds of the interaction of SiO_2_·0.7H_2_O and Zn(H_2_PO_4_)_2_·2H_2_O hydrates with other components. According to the reaction equations (Table 1), 5.7 mol of water (9.2 wt.%) should be released in the reaction mixture at x = 1, while 9.4 mol of water (14.3 wt.%) should be released at x = 2. At x = 1, the released water participates in the formation of a calcium hydroxide phase (Figure 1). It is possible that at x = 2, there is more water than is involved in the hydroxide formation. Water molecules present in the reaction mixture can be both sorbed by the surface of the particles and incorporated into the HA crystal lattice [21,22]. According to Table 1, at x = 2, apatite with composition Ca_8.0_Zn_2.0_(PO_4_)_4.0_(SiO_4_)_2.0_ should crystallize. In this case, there are no hydroxyl groups in the crystal lattice (otherwise appearing due to charge compensation). This means that an apatite hydroxyl channel contains only hydroxyl vacancies. These vacancies can be occupied by water molecules owing to a large channel diameter [23]. The incorporation of a large number of water molecules into the apatite lattice hinders the formation of HA crystals as the electrostatic attraction between the ions is destroyed. This leads to the presence of an amorphous phase at high concentrations.

**Figure 2:**
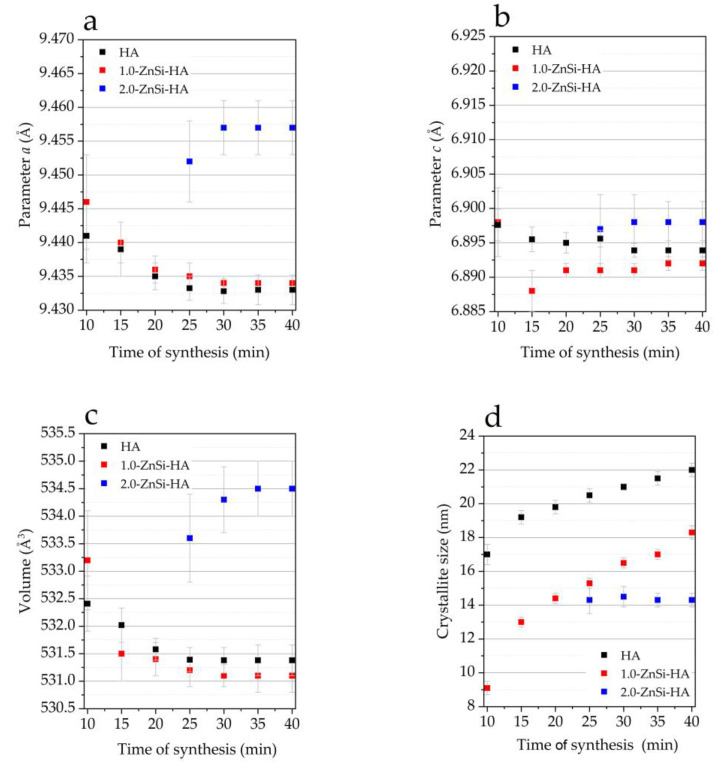
Evolution of lattice parameters (**a**,**b**), volume (**c**), and crystallite size (**d**) of HA phase during synthesis of samples with different dopant concentration.

Despite the disappearance of the reflections of the initial reagents after short treatment times, the parameters and unit cell volume of the HA phase continued to change up to 35 min (Figure 2). Therefore, the optimal time of the mechanochemical synthesis of HA co-substituted with zinc and silicate is 35 min.

Figure 2 shows that the lattice parameter *a* and unit cell volume of 2.0-ZnSi-HA increased with the time of synthesis, showing trends opposite to those observed for 1.0-ZnSi-HA. The incorporation of large amount of water molecules into the hydroxyl channel, as mentioned above, increased the channel diameter, which led to an increase in the basal parameters (*a* and *b*) and an increase in the unit cell volume. 

### 3.2. Mechanochemical Synthesis of ZnSi-HA Samples

**Figure 3:**
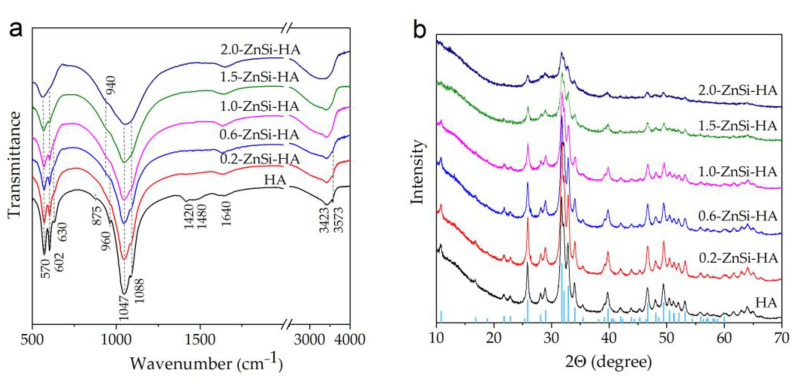
FTIR spectra (**a**) and XRD patterns (**b**) of samples with different dopant concentrations after 40 min of treatment.

Figure 3a shows the FTIR spectra of HA co-substituted with zinc and silicate together with that of the nonsubstituted HA. The latter shows the absorption bands corresponding to the HA structure: the absorption bands of the phosphate ion (570, 602, 960, 1047, and 1088 cm^−1^) and of the hydroxyl group (630 and 3573 cm^−1^) [23]. In addition, small-intensity absorption bands at 875, 1420, and 1480 cm^−1^ were observed in the spectrum of the HA sample. According to Ref. [24], these bands belong to the carbonate ion in the position of the phosphate group in the HA structure. Upon substitution, the broadening of the absorption bands of the phosphate groups and disappearance of the OH-group bands are observed. The absorption bands of the carbonate group are absent in the spectra of the co-substituted HA samples. In addition, the spectra of the co-substituted HA demonstrate an absorption band at a wavelength of 940 cm^−1^. This band was detected in the FTIR spectra of Si-HA samples and attributed to silicate ions [11]. The wide absorption bands at wavelengths of 1640 and 3423 cm^−1^ were associated with vibrations in the water molecules. Figure 3a shows that the shape of absorption band at 3423 cm^−1^ gradually changes. The band becomes broader and its integral intensity increases with increasing dopant concentration. In the sample with the maximal amount of released water, namely 2.0-ZnSi-HA, this band was more pronounced than for other samples, and the phosphate ion bands were poorly resolved.

**Figure 4:**
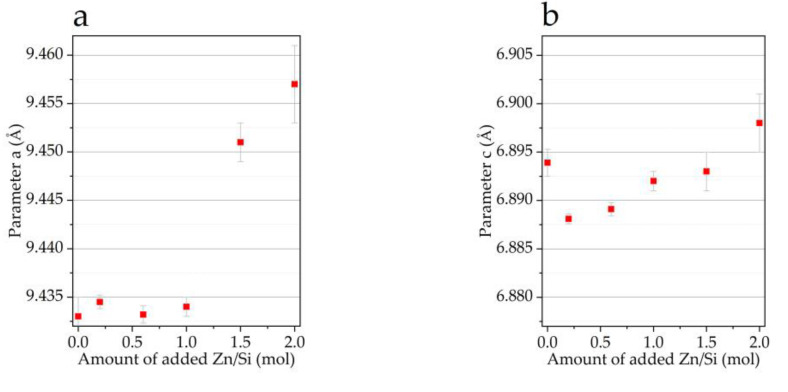

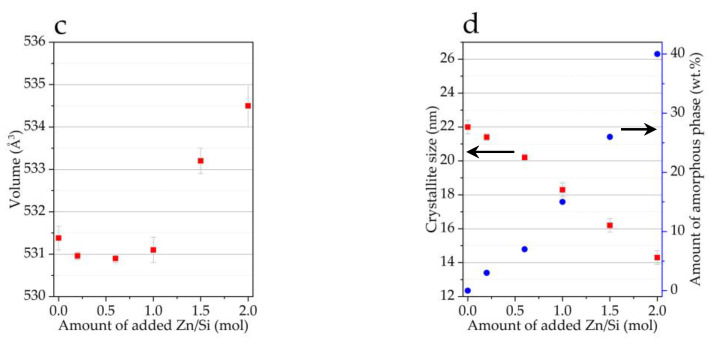
Dependence of lattice parameters (**a**,**b**), volume (**c**), and crystallite size of the HA phase and amount of the amorphous phase (**d**) on the dopant concentration. The duration of synthesis for all samples was 40 min. The left arrow indicates the assignment of red squares to the “Crystallite size” axis, and the arrow right indicates the assignment of blue dots to the axis “Amount of amorphous phase”.

The XRD patterns of the as-synthesized powders with different concentrations of dopants treated for the same time (40 min) are presented in Figure 3b. All these patterns show the formation of only one crystal phase, namely the HA phase (PDF card 40-11-9308). An increase in the concentration of dopants led to a decrease in the intensity of reflections and the appearance of an amorphous phase, which is clearly visible in the diffraction pattern of the 2.0-ZnSi-HA sample. The results of the quantitative analysis indicated that the concentration of the amorphous phase in the samples increased with the dopant concentration (Figure 4d).

The lattice parameters and crystallite size of the obtained compounds were refined by the Rietveld method (Figure 4). The lattice parameters have a complex dependence on the concentrations of the substituents. The volume of the unit cell and the lattice parameters decreased as x increased from 0 to 0.6 and then increased with a further increase in x. This may have been due to the fact that at low concentrations of the substituents, a smaller ionic radius of zinc in comparison with that of calcium played a role [25]. At high concentrations, this contribution was insignificant, and the difference between the ionic radii of the silicate and phosphate played the main role, causing lattice expansion. In addition, the introduction of water molecules into the lattice can lead to an increase in the volume of the cell [21], which is consistent with the data in Table 1. It can be seen that with an increase in the concentration of the introduced substituent ions, the concentration of the released water increased. Both the released water molecules and the substituent ions themselves complicated the formation of the HA crystal lattice, so the crystallite size decreased with increasing x. Based on data presented in Figure 4, we consider the substitution limit for the mechanochemical method to be x = 1.0. At higher concentrations of the dopants, water molecules were incorporated into the lattice of the substituted HA.

**Figure 5:**
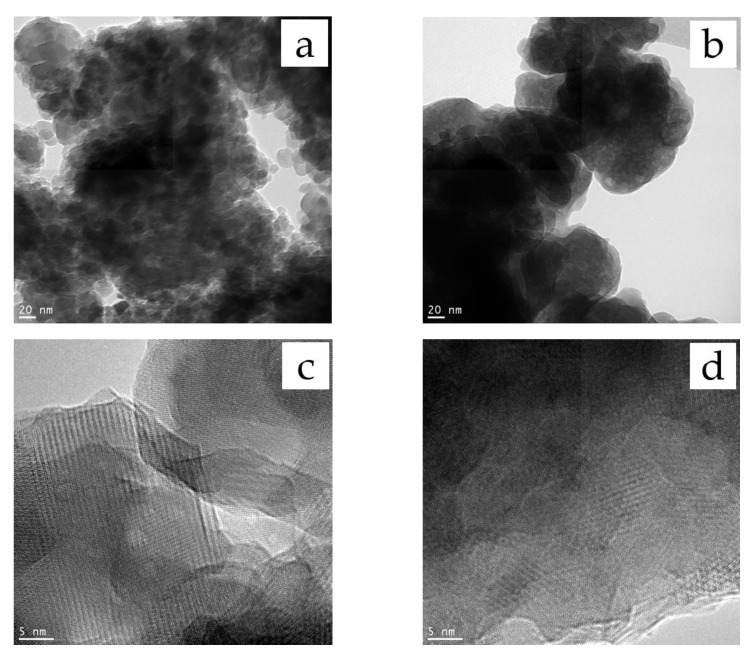
TEM (**a**,**b**) and HRTEM (**c**,**d**) images for HA (**a**,**c**) and 2.0-ZnSi-HA (**b**,**d**) samples.

**Figure 6:**
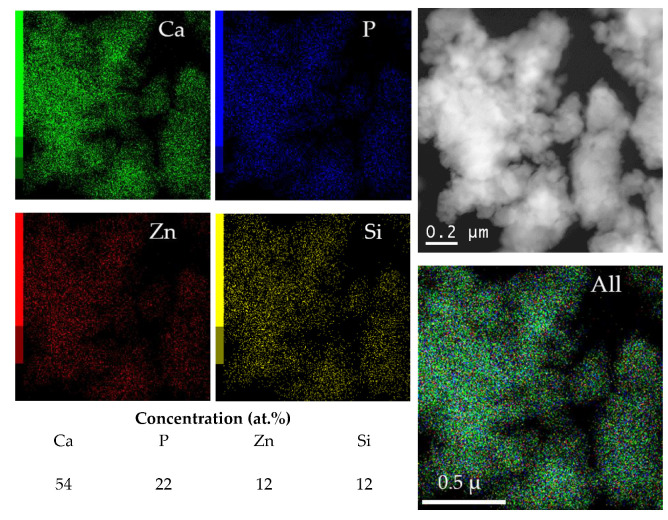
Distribution map of elements across particles in 2.0-ZnSi-HA sample. Data obtained by TEM measurement.

As seen in Figure 5, the introduction of dopants changed the particle morphology. Nonaggregated particles smaller than 20 nm in size were observed in the unsubstituted HA sample. At x = 2, the particles formed aggregates ~100 nm in size. Although the XRD data indicated the presence of a large amount of an amorphous phase in the 2.0-ZnSi-HA sample, HRTEM showed that all particles were crystalline. This discrepancy can be explained by the fact that amorphous calcium phosphates are prone to crystallization under an electron beam due to intensive heating during HRTEM measurements [22]. Figure 6 shows that the distribution of the elements in the particles of 2.0-ZnSi-HA was uniform. Their concentrations were close to the expected concentrations (Table 2). Despite the fact that during the synthesis of 2.0-ZnSi-HA, the amount of released water (causing agglomeration of the particles) was 4.7 times that released during HA synthesis (Table 1), the reagents were efficiently mixed.

Table 2 presents the results of the ICP-AES analysis of the cosubstituted HA, which show that with an increase in the concentration of the introduced dopants, the measured concentration of the elements increased. There was also good agreement with the expected compositions (within the measurement error). Taking into account the results of the elemental analysis performed during the TEM measurements (Figure 6), it can be assumed that there may be a slight overestimation in the ICP data for silicon.

### 3.3. In Vitro Investigations

Because the double substitution limit for zinc and silicon was set to x = 1.0, the in vitro studies were carried out only on the HA, 0.2-ZnSi-HA, 0.6-ZnSi-HA, and 1.0-ZnSi-HA samples. 

**Figure 7:**
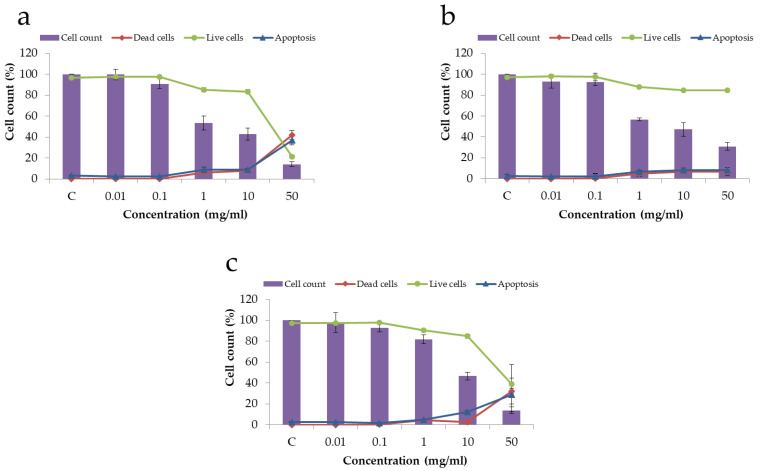
Cell viability of Hek293 cells incubated with HA (**a**), 0.2-ZnSi-HA (**b**), or 1.0-ZnSi-HA (**c**) for 48 h.

The cytotoxicity of the samples was tested against the Hek293 cell line. When Hek293 cells were incubated with different concentrations of the tested compounds for 48 h, good cell viability was observed up to a concentration of 1 mg/mL, as shown in Figure 7. A slight cytotoxic effect appeared at a concentration of 1 mg/mL for all investigated samples and increased at higher concentrations of the powder. By comparing the samples with different concentrations of the substituent ions, we concluded that at a substituent concentration of x = 1 (sample 1.0-ZnSi-HA), a slight increase in the cell viability wad observed at a powder concentration of 1 mg/mL. At a powder concentration of 10 mg/mL, the cell count decreased and became the same for all samples (around 45%). At the maximum HA and 1.0-ZnSi-HA concentration studied (50 mg/mL), a significant decrease in the cell count and an increase in dead cells together with apoptotic cells were observed. This toxic effect on the cells could be associated with hypoxia and nutrient deficiency caused by particle sedimentation over the cells [16,26]. Nevertheless, despite the overall low number of cells observed, the 0.2-ZnSi-HA sample contained the same levels of live, dead, and apoptotic cells as observed at a lower powder concentration, indicating a positive effect of the low concentrations of the dopants on cell viability.

The obtained data on the relatively low toxicity of HA and HA cosubstituted with zinc and silicate and increased cell viability are in agreement with the data in the literature [27,28]. Thus, we concluded that the obtained ZnSi-HA materials are nontoxic. Cytotoxic properties were observed only at a concentration of the powder in solution of 50 mg/mL, which was most likely due to hypoxia and nutrient deficiency caused by particles’ sedimentation over the cells. The presence of a small amount of substituents (x = 0.2) in HA reduced the cytotoxicity of the latter at this concentration of the powder in solution. At the higher concentration of substituents (x = 1.0), the cytotoxicity was again at the level of that of the unsubstituted HA.

A standard MTT assay was performed to determine the powder cytotoxicity on human MG-63 osteoblasts. Table 3 shows the results of the cell incubation with the HA powders for 4 days. The results showed that none of the studied samples had a cytotoxic effect. Moreover, doping of HA with Zn and Si ions at a degree of substitution of x = 0.2 led to a significant increase in the growth of osteoblasts. This result is consistent with previously reported data [29]. At a higher ion concentration, the osteoblasts grew at a lower rate. For a substituent concentration of x = 0.6, the optical density value was even lower than that of the control.

**Figure 8:**
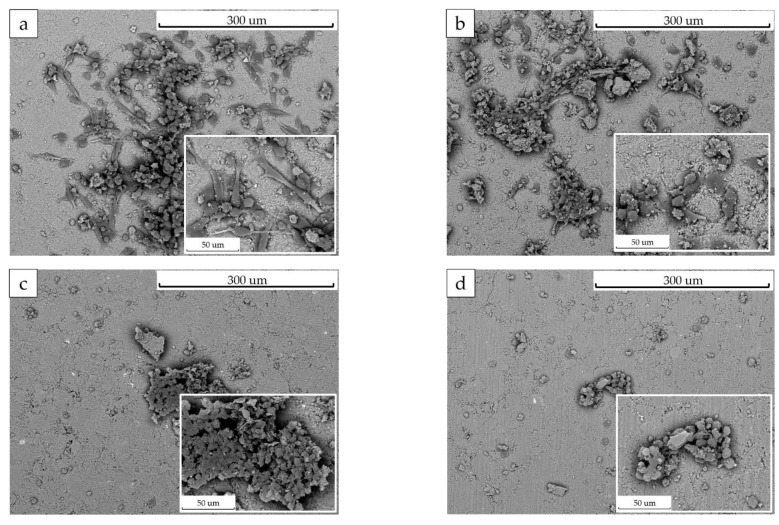
SEM images of surface of ZnSi-HA pellets after 7-day incubation of MG-63 cells: (**a**) HA, (**b**) 0.2-ZnSi-HA, (**c**) 0.6-ZnSi-HA, and (**d**) 1.0-ZnSi-HA.

For the bone cell proliferation assessment, the osteoblastic MG-63 cells were cultured on the surface of pellets made of HA with different concentration of the substituents. The SEM micrographs of the osteoblasts after 7 days of culturing on the pellets are shown in Figure 8. It can be seen that cells were observed in large numbers on the HA (Figure 8a) and 0.2-ZnSi-HA (Figure 8b) pellets. On the 0.6-ZnSi-HA (Figure 8c) and 1.0-ZnSi-HA pellets (Figure 8d), the number of the attached cells was much smaller. A comparative analysis of the cell morphology indicated that well-attached spindle cells were observed only on the HA (Figure 8a) and 0.2-ZnSi-HA (Figure 8b) samples, with the highest proliferation found in the unsubstituted HA. Because the samples consisted of particles of the same size, the effect of the particle size on the cell growth was excluded. Obviously, there was a toxic effect of the substituent ions, most likely zinc. This observation agrees with results obtained by other authors in studies of the single substitution by zinc. Decreases in the cell proliferation [30], biocompatibility, and osteoconductivity [26] for HA containing more than 0.2 mol of Zn were observed. This effect did not occur in silicon-substituted HA [8]. Thus, it can be concluded that the acceptable degree of cosubstitution is x = 0.2. At high concentrations of zinc and silicon in the material, the attachment and proliferation of MG-63 cells become weak. 

The solubility studies of the samples of the unsubstituted HA and 0.2-ZnSi-HA showed that the solubility of HA cosubstituted with zinc and silicate was approximately twice that of the unsubstituted HA (Table 4). We concluded that the introduction of zinc and silicon ions accelerates the bioresorption of the material.

## 4. Conclusions

In this study, we found that mechanochemical synthesis can be used to obtain substituted HA containing zinc and silicon ions in its structure (cosubstituted HA). We found that the formation of the crystal lattice of the cosubstituted HA was complete after 35 min of treatment of the reaction mixtures in a high-energy planetary ball mill. The limit of possible substitution was found to be x = 1.0. With a further increase in the substituent concentration, a significant increase in the unit cell volume was observed. As the dopant concentration increased, the crystallite size of the product decreased, while the concentration of the amorphous phase increased. In the FTIR spectra of the cosubstituted HA, in addition to the absorption bands of the phosphate and hydroxyl groups, the presence of bands of silicate ions was observed. The TEM studies confirmed the uniform distribution of the elements in the particles of the synthesized material.

The in vitro studies revealed that the introduction of zinc and silicon ions into the HA structure at a concentration of x = 0.2 improved the biocompatibility, decreased the cytotoxicity, and increased the solubility of the material. This composition is recommended for further in vivo investigations of the biodegradation of a granulated material or bioresorbable scaffold. Samples with a higher concentration of the substituent ions can be used as a biologically active additive in the development of bioresorbable multicomponent materials. The cosubstituted apatite with high concentrations of the substituents can also be used as a source material for producing calcium phosphate coatings by microarc oxidation, magnetron sputtering, or other deposition method, in which the ionization of the sprayed material takes place. In this case, the concentrations of the doping elements in the coating may differ from those in the initial material due to the different deposition rates of different ions.

## Figures and Tables

**Table 1:** Ratio of the initial reagents and expected product of the mechanochemical synthesis.

Degree of Substitution (x)	Sample Designation	Equation of the Expected Chemical Reaction
0	HA	6.0 CaHPO_4_ + 4.0 CaO $\overset{MS}{\to }$ Ca_10_(PO_4_)_6_(OH)_2_ + 2 H_2_ O
0.2	0.2-ZnSi-HA	5.4 CaHPO_4_ + 4.4 CaO + 0.2 Zn(H_2_PO_4_)_2_·2 H_2_O + 0.2 SiO_2_·0.7 H_2_O $\overset{MS}{\to }$ Ca_9.8_Zn_0.2_(PO_4_)_5.8_(SiO_4_)_0.2_(OH)_1.8_ + 2.74 H_2_O
0.6	0.6-ZnSi-HA	4.2 CaHPO_4_ + 5.2 CaO + 0.6 Zn(H_2_PO_4_)_2_·2 H_2_O + 0.6 SiO_2_·0.7 H_2_O $\overset{MS}{\to }$ Ca_9.4_Zn_0.6_(PO_4_)_5.4_(SiO_4_)_0.6_(OH)_1.4_ + 4.22 H_2_O
1.0	1.0-ZnSi-HA	3.0 CaHPO_4_ + 6.0 CaO + 1.0 Zn(H_2_PO_4_)_2_·2 H_2_O + 1.0 SiO_2_·0.7 H_2_O $\overset{MS}{\to }$ Ca_9.0_Zn_1.0_(PO_4_)_5.0_(SiO_4_)_1.0_(OH)_1.0_ + 5.7 H_2_O
1.5	1.5-ZnSi-HA	1.5 CaHPO_4_ + 7.0 CaO + 1.5 Zn(H_2_PO_4_)_2_·2 H_2_O + 1.5 SiO_2_·0.7 H_2_O $\overset{MS}{\to }$ Ca_8.5_Zn_1.5_(PO_4_)_4.5_(SiO_4_)_1.5_(OH)_0.5_ + 7.55 H_2_O
2.0	2.0-ZnSi-HA	8.0 CaO + 2.0 Zn(H_2_PO_4_)_2_·2 H_2_O + 2.0 SiO_2_·0.7 H_2_O $\overset{MS}{\to }$ Ca_8.0_Zn_2.0_(PO_4_)_4.0_(SiO_4_)_2.0_ + 9.4 H_2_O

**Table 2:** Results of ICP-AES analysis of the as-synthesized samples.

Sample	Concentration (at.%)							
	Composition of Synthesized Materials				Expected Composition			
	Ca	P	Zn	Si	Ca	P	Zn	Si
HA	63.9 ± 8.3	36.1 ± 3.0	-	-	62	38	-	-
1.0-ZnSi-HA	56.1 ± 3.5	29.1 ± 3.5	6.4 ± 1.5	8.4 ± 1.5	57	31	6	6
2.0-ZnSi-HA	50.0 ± 2.8	22.6 ± 1.7	11.5 ± 0.5	15.9 ± 3.5	51	25	12	12

**Table 3:** MTT assay results after 4-day incubation of MG-63 cell with HA powders. Optical density of formazan inversely correlated with cell death.

Sample	Optical Density of Formazan
HA	0.584 ± 0.06
0.2-ZnSi-HA	0.600 ± 0.08
0.6-ZnSi-HA	0.477 ± 0.09
Control	0.541 ± 0.05

**Table 4:** Results of elemental analysis of solutions after soaking powders in water.

Time (Day)	Concentration (ppm)					
	HA		0.2-ZnSi-HA			
	Ca	P	Ca	P	Si	Zn
1	2.8 ±0.4	0.7 ± 0.1	5.0 ± 0.6	2.1 ± 0.3	0.7 ± 0.1	0.13 ± 0.02
3	3.6 ±0.5	2.0 ± 0.3	3.3 ± 0.5	1.4 ± 0.2	1.3 ± 0.2	0. 06 ± 0.01
5	1.7 ± 0.4	1.0 ± 0.1	2.4 ± 0.4	1.1 ± 0.2	0.7 ± 0.1	0.08 ± 0.01

## Data Availability

The raw/processed data required to reproduce these results are included in the Section 2.
